# Chemical Composition and Related Properties of Lime (*Tilia cordata* Mill.) Bark and Wood as Affected by Tree Growth Conditions

**DOI:** 10.3390/ma15114033

**Published:** 2022-06-06

**Authors:** Władysław Kusiak, Jerzy Majka, Magdalena Zborowska, Izabela Ratajczak

**Affiliations:** 1 Department of Forest Engineering, Faculty of Forestry and Wood Technology, Poznań University of Life Sciences, Wojska Polskiego 71C, 60-625 Poznań, Poland; wladyslaw.kusiak@up.poznan.pl; 2 Department of Wood Science and Thermal Techniques, Faculty of Forestry and Wood Technology, Poznań University of Life Sciences, Wojska Polskiego 38/42, 60-637 Poznań, Poland; jerzy.majka@up.poznan.pl; 3 Department of Chemical Wood Technology, Faculty of Forestry and Wood Technology, Poznań University of Life Sciences, Wojska Polskiego 38/42, 60-637 Poznań, Poland; magdalena.zborowska@up.poznan.pl; 4 Department of Chemistry, Faculty of Forestry and Wood Technology, Poznań University of Life Sciences, Wojska Polskiego 75, 60-625 Poznań, Poland

**Keywords:** environmental stress, chemical elements, FAAS, hygroscopicity, dynamic vapor sorption, equilibrium moisture content, sorption hysteresis, GDW model, multi-factor ANOVA

## Abstract

*Tilia cordata* Mill. is a favourite tree used in urban spaces. For this reason, it is important to know its sensitivity to environmental stress, which is particularly burdensome for vegetation in urban spaces. The aim of the study was to investigate the properties necessary to control the growth of these trees and their subsequent use, i.e., chemical properties (percentage contents of cellulose, holocellulose, lignin, pentosans and substances soluble in NaOH and EtOH) as well as the chemical elements (K, Na, Mg, Ca and Fe, Zn, Cu, Pb, Cd, B, Ni, Cr, Al, As and Hg) and selected hygroscopic properties (hysteresis and sorption isotherms). Trees of *Tilia cordata* Mill. growing in environments exposed to environmental stress of varying severity were examined. Regardless of the growth conditions, in terms of its chemical composition, bark differs significantly from wood, showing twice the contents of soluble substances in NaOH and lignin and half the content of polysaccharides. Growth conditions clearly affect the range of selected chemical components in bark, e.g., substances soluble in ethanol, cellulose, or lignin. The main inorganic elements in bark and wood are Na, K, Ca, Mg and Zn. In bark, a relationship was found between the content of most chemical elements and differing environmental growth conditions. It was shown that environmental stress influenced the hygroscopic properties of wood and bark, which are a consequence of the percentage of chemical components.

## 1. Introduction

Small-leaved lime is a tree species investigated in a large number of studies and as such is discussed in numerous publications. When presenting the history of small-leaved lime in the Białowieża Forest, Ref. [1] cited many valuable historical articles [2,3,4], indicating the considerable importance of this species. *Tilia cordata* Mill. is a favourite tree used in urban landscapes. Small-leaved lime exhibits high aesthetic qualities as well as shade tolerance and frost resistance. It is able to withstand temperature changes and simultaneous water deficit in the soil [5]. Due to their high resistance, lime leaves have been used as a bioindicator of environmental pollution in urban areas. In turn, Sadowiec and Gawroński [6] studied selected lime species (*Tilia* sp.) for air phytoremediation from dust pollutants.

*Tilia* continues to be regarded as a useful, although not valuable, timber, and is used in applications such as turnery [7]. *T. cordata* and *T. platyphyllos* can be worked easily and has been a highly favoured material for carving [8].

Interest in the utilisation of bark dates back many centuries, when it was a valuable raw material of economic importance, used to produce tannins and pigments. Young bark of selected species is highly prized because of its medicinal properties due to presence of various alkaloids (e.g., betulin, taxine, aesculin, fraxin and salicin). Bark is a valuable raw material for the pharmaceutical and cosmetics industries, as a source of fuel, or in horticulture as a substrate for composting [9]. The role of bark as a biocomposite in the production of insulation materials was extensively discussed in a review paper by Gianntos et al. [10]. The applicability of bark in biomonitoring was indicated by Kosiorek et al. [11], Krutul et al. [12] and Pavlović et al. [13]. Barks of lime are sensitive to ambient air and thus they may be used as bioindicators to determine the level of heavy metal pollution [14,15]. Bark surrounding the tree from the outside constitutes a protective layer against the influence of abiotic and biotic factors (insects and fungi). Bark differs considerably from xylem in terms of the contents of lignin, carbohydrates or suberin. Compared to xylem, bark contains higher amounts of minerals, extractives and pectin substances, while its contents of cellulose, pentosans and lignin are typically lower [16]. This is confirmed by studies showing that, in comparison to xylem, bark contains greater amounts of minerals, substances soluble in hot water as well as lignin, at a markedly lower content of cellulose [14]. Contents of extractives in bark depend on the species, tree age, habitat conditions and height of the stem from which it was collected. In the case of lime, a considerable proportion of bark is composed of phloem, which is adjacent to xylem and promotes the transport of assimilates [17].

In the case of *Tilia cordata* Mill., its xylem, bark, flowers, fruits and leaves are of economic importance. Various definitions of bark, composed of the inner layer (phloem) and the outer layer, i.e., cork cambium (phellogen), are used [18]. Th literature concerning the chemical composition of bark is extensive [14], particularly in relation to its extractive components [19,20,21].

A comparison of sorption data for mature and juvenile lime (*Tilia* sp.) wood was presented in the publication by Majka and Olek [22]. It was found that a significantly lower equilibrium moisture content (*EMC*) was observed for mature lime wood as compared to juvenile wood. In the above-mentioned study, the contents of the monolayer and multilayer water were separately determined using the Hailwood-Horrobin model [23], according to Simpson [24], for different sorption phases. It was found that the maximum value of the monolayer water content was practically the same for juvenile and mature wood. The maximum value of the multilayer water content was lower for mature wood by ca. 0.04 kg/kg as compared to the juvenile wood during the adsorption and second desorption.

In a more recent study, Kusiak et al. [25] compared properties of lime (*Tillia cordata* Mill.) trees growing in different conditions, i.e., areas varying in their degrees of environmental stress. It was stated that environmental stress affected sorption, which was a consequence of the chemical composition. In juvenile and mature lime wood, hygroscopicity is enhanced by an adverse environmental stress impact. A major environmental stress results in an increased content of extractives, which in turn leads to increased wood hygroscopicity. Moreover, the analysis of the sorption phenomenon performed applying the coefficients of the GAB model showed that as a result of increasing environmental stress, the monolayer water was bound more strongly to the primary sorption sites. The bark–water relations and moisture transport in the bark of beech (*Fagus sylvatica*) and spruce (*Picea abies*) were studied by Rémond et al. [26]. The data fitting to the adsorption–desorption curves calculated applying the Hailwood-Horrobin model shows that beech bark has almost the same hygroscopicity as wood, whereas the bark of spruce is more hygroscopic than wood over the entire *RH* range. The sorption hysteresis is approximately the same for both barks, while it is more pronounced between spruce and beech woods. It has also been shown that the bark of Red Oak and Yellow Poplar dries more intensively than sapwood and heartwood [27]. To the best knowledge of the authors, no published results are available concerning the hygroscopic properties of lime bark.

Resistance to adverse environmental conditions, including heavy metal pollution, water deficit and CO_2_ emissions make lime a species suitable for urban landscaping. Moreover, lime trees exhibit a considerable capacity to accumulate pollutants in urban agglomerations, characterised by a high level of environmental stress. In view of the above, this study aims to understand the role of trees’ growing conditions under different environmental conditions on selected properties of the bark and wood of *Tilia cordata* Mill.

## 2. Materials and Methods

### 2.1. Sampling

**Figure 1:**
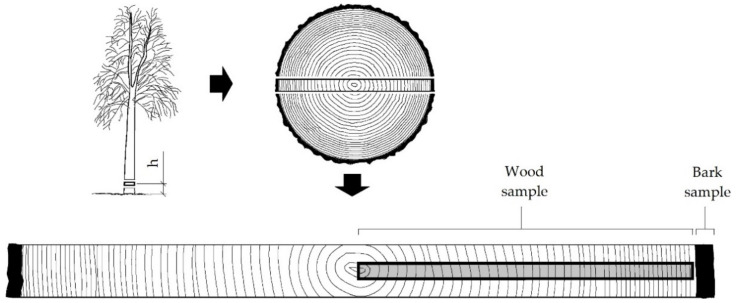
The scheme of sampling (h—breast height diameter (1.3 m)).

The study was carried out on *Tilia cordata* Mill. bark and wood of trees from the Wielkopolska-Pomeranian natural-forest region, characterised, among other things, by lowland character, mild, warm climate with low amounts of precipitation, and the dominance of coniferous forest habitats. The average height of the lime trees was 19–21 m. There were divided into three categories in terms of the environment of their growth conditions. Detailed characteristics of the sampling sites are described in the publication by Kusiak et al. [25]. Trees growing in the forest were exposed to the lowest environmental stress, trees growing by the road were exposed to medium environmental stress, and trees growing in an urban agglomeration were exposed to the highest level of environmental stress. The seasoned samples in the form of a disc of 60 mm (in longitudinal anatomical direction) were collected (at breast height) along the North–South axis. The strips were radially oriented and included all the growth rings from the pith to bark. Figure 1 presents the scheme of sample preparation. The prepared bark and wood samples were ground in a Fritsch Pulverisette 15 laboratory mill (Fritsch GmbH, Weimar, Germany). Each prepared sample was fractioned and divided into two subsamples. The 0.5–1.0 mm fraction (mass ca. 50 g) and the 0.125–0.250 mm fraction (mass ca. 8 mg) were used to analyse the chemical composition and sorption, respectively.

### 2.2. Chemical Analysis

The bark and wood samples were used in the chemical analyses. The prepared raw samples were ground in a Fritsch Pulverisette 15 laboratory mill (Fritsch GmbH, Weimar, Germany), and the 0.5–1.0 mm fraction (mass ca. 50 g) was used.

### 2.3. Chemical Composition of Bark and Wood

The percentage of carbohydrates, i.e., holocellulose, cellulose and pentosans, was determined by chlorite methods [28], using NaClO_2_, Seifert [28] with a mixture of acetylacetone, 1,4-dioxane and hydrochloric acid, and TAPPI T 223 cm-01 [29] with hydrochloric acid and floroglucinol. The lignin content was determined according to the TAPPI method T 222 om-06 [30] using 72% sulfuric acid. The extracts soluble in organic solvents were determined according to the T 204 cm-97 standard [31] using EtOH. The solubility in 1% NaOH was determined according to T 212 om-02 [32]. All chemical analyses were repeated in triplicate for each bark and wood sample. The maximum standard deviation of the results in each test was assumed as an indicator of the measurement error.

### 2.4. Determination of Chemical Elements

The ground samples (0.5000 g) were mineralised with HNO_3_ (Avantor Performance Materials, Gliwice, Poland) in the microwave mineralization MARSXpress system (CEM Corporation, Matthews, NC, USA). Contents of chemical elements in the samples were analysed by means of AAS (Atomic Absorption Spectroscopy) using a Spectra 280 AA spectrometer (Agilent Technologies, Santa Clara, CA, USA). The calibration curve was prepared from the serial dilution of the standard element (K, Na, Mg, Ca and Fe, Zn, Cu, Pb Cd, B, Ni, Cr, Al, As and Hg) solution (Sigma-Aldrich, Saint Louis, MO, Germany). The correctness of the method was verified using the certified reference material NCS DC 73350 (NACIS, Shanghai, China). The final results were average values of three simultaneous measurements.

### 2.5. Sorption Experiments

Prior to the experiments, the prepared bark and wood samples (0.125–0.250 mm fraction) were stored in a desiccator over phosphorus pentoxide (P_2_O_5_) in order to obtain the near-dry state. The initial mass of each investigated sample was 8 ± 0.5 mg. The sorption experiments were performed using the dynamic vapor sorption apparatus (DVS Advantage 2, Surface Measurement Systems, London, UK) at a temperature of 20 °C. Additionally, the DVS experiments started with the sample equilibration in dry gas (nitrogen) in order to obtain the dry state. The *EMC* values were measured for 12 levels of air relative humidity (*RH*) at a flow rate of 150 cm^3^/s. The scheduled *RH* ranged from 0% to 95% (adsorption mode) and back to 0% (desorption mode). The *EMC* values were measured for 12 *RH* levels, separately for adsorption and desorption phases. In the present study, a sample approached equilibrium at a given air *RH* value when the mass change was less than 0.0005%/min over a 10 min window. The measured *EMC* values were used to obtain adsorption and desorption isotherms for all investigated options of tissue and stress environments and parametrised with the GDW model [33,34]. The GDW model is given by the following equation:

(1) $$EMC=\frac{m\cdot K\cdot RH}{\left(1+K\cdot RH\right)}\cdot \frac{1-k\cdot \left(1-w\right)\cdot RH}{\left(1-k\cdot RH\right)}$$

where *m* kg/kg—maximum monolayer water content (water bound to the primary sorption sites); *K*—kinetic constant related to sorption on the primary sites; *k*—kinetic constant related to sorption on the secondary sorption sites; *w*—ratio of water molecules bound to the primary sites and converted into the secondary sites. The least-square method was used to estimate the coefficients of Equation (1). The sorption hysteresis was quantified by the maximum difference of *EMC* for desorption and adsorption (Δ*EMC*) and by the hysteresis loop (*H*) and its relative change (δH) [35].

### 2.6. Statistical Analysis

The results of the experiment were analysed using the STATISTICA 13.3 software (TIBCO Software Inc., Palo Alto, CA, USA). A two-way analysis of variance (ANOVA) was performed to determine whether the tissue type and the environment affected the chemical composition of examined lime bark and wood. Moreover, the post hoc HSD Tukey’s test was applied to test the significance of differences between average values of the mean contents of structural components and chemical composition. Significance was established at *p* < 0.05.

## 3. Results

### 3.1. Chemical Analysis

#### 3.1.1. Chemical Composition of Bark and Wood

In the case of bark and wood, chemical composition percentage contents were determined for the main components, i.e., holocellulose [28], cellulose [28], pentosanes [29] and lignin [30] as well as components soluble in 1% NaOH [31] and in ethanol [31]. Two-way ANOVA indicated a significant (*p* < 0.05) effect of tissues on contents of each presented component. Further, the environment significantly (*p* > 0.05) influenced contents of lignin and substances soluble in alcohol (Table S1). Interaction of the variables (a × b) was also found statistically significant for the contents of cellulose, lignin and substances soluble in NaOH.

Table 1 shows contents of the assayed bark and wood chemical components. For bark of trees from the environments of low, medium and high environmental stress, the total carbohydrate content (holocellulose) ranged from 57.1 to 60.0%, with the differences being non-significant. In wood, the holocellulose content was about 40% higher. The cellulose content in bark ranged from 27.8 to 33.4%. The highest cellulose amount was recorded for the bark of trees growing in the high stress environment. In this wood the cellulose content was also on average about 40% higher. The highest cellulose content was found for the trees growing under low stress. The content of pentosanes, the best hygroscopic component of wood, varied in the investigated tree barks from 12.3 to 13.4%, while in wood it was twice as high. No significant differences were observed either in bark or wood. Compared to the literature data, i.e., 19.7–20.4% [36], the determined percentage content was slightly higher. For all the carbohydrates analysed above, their content was higher in wood than in bark. This observation is consistent with literature reports [37].

Lignin content in the bark of the compared trees ranged from 41.0 to 50.0%. The differences were found to be significant for all the results. The greatest lignin amount was determined in bark of the trees growing under medium environmental stress. In wood, the lignin percentage content was two times lower. The same can be said for substances soluble in 1% NaOH. In the case of this heterogenous group of compounds (hemicelluloses, low-polymerised lignin, amorphous cellulose, fats and waxes), the differences between bark and wood were also evident, with greater amounts recorded in bark. For both tissues, significant differences were found for the trees growing in the forest (low environmental stress). Substances soluble in EtOH are the components from the group of lipid compounds, i.e., mainly resins, fats and waxes. Their content in bark ranged from 11.8 to 16.4%, whereas in wood it was slightly lower and ranged from 6.5 to 10.3%. For bark, the greatest amount was recorded for trees growing in an urban agglomeration (high environmental stress).

It is worth noting that for the bark of trees growing in areas that differ in terms of environmental stress, the percentage contents of substances soluble in EtOH, lignin and cellulose differed statistically. No such difference in the statistical percentage of these components was found for wood of trees growing in these areas. It can be concluded that bark shows a stronger interaction (i.e., changes in the chemical composition) with the surrounding environment than wood.

#### 3.1.2. Determination of the Chemical Elements of Bark and Wood

Table S2 presents the results of two-way ANOVA for the investigated chemical composition groups of elements, i.e., K, Na, Mg and Ca. The results of this study indicate a significant effect of tissue type on the content of individual components (at *p* < 0.05). Statistically significant interactions of factors (a × b) were also found for all the analysed variants of the chemical composition in *Tilia cordata* Mill. bark and wood.

Contents of potassium, sodium, magnesium and calcium (Table 2) both in the bark and wood of lime varied within a wide range of values, with the differences between the results being statistically significant. In the bark of the investigated trees, the highest potassium levels were recorded for trees growing under low environmental stress (in the forest), while they were lowest in the bark from trees growing under high environmental stress (urban agglomeration). The greatest potassium content for wood samples was found for a tree growing under high environmental stress, while it was lowest for a tree growing under low stress. Only in the case of Ca was it observed that the percentage content of this element in bark and wood was related with the intensity of environmental stress. The greatest amount of calcium both in bark and wood was from a tree growing in an urban agglomeration (under high stress), whereas it was smallest in a tree growing in the forest (under low stress).

This study showed a dependence between very high contents of potassium and calcium in bark of a tree growing under low environmental stress, while low contents of heavy metals, e.g., iron, zinc, lead, cadmium and chromium. In wood samples from a tree growing under high environmental stress, in which very high levels of potassium and calcium were recorded, the highest concentrations of cadmium, nickel and chromium were also reported.

Table S3 presents the results of two-way ANOVA for the investigated groups of the chemical elements (Fe, Zn, Cu, Pb, Cd, B, Ni, Cr, Al, As and Hg). The analysis results indicate significance of the effect of tissue type on the contents of each of the presented components (for *p* < 0.05). Statistically significant interactions of factors (a × b) were also seen for all the analysed cases of the investigated chemical composition in *Tilia cordata* Mill. (bark and wood).

Based on the results given in Table 3 a and b, the contents of more than a half of elements, i.e., Zn, Pb, Cd, Cr, Ni, As and Hg, in lime bark increased with the growing intensity of environmental stress. The lowest amounts of these elements were recorded in the bark of trees growing under low stress, while the levels were highest in the bark of trees growing under high stress conditions. In the case of wood, the increase in these contents with the growing intensity of environmental stress was observed for only two elements, i.e., Cd and Cr. A reduction in the contents of the elements with an increase in stress intensity was detected in bark in only one case (for B), while in wood it was in two cases (B and Al). Thus, in bark, only for 2 out of the 11 elements (here it was Fe and Cu), no effect of environmental stress was observed. In contrast, in wood it was observed for seven elements. This is consistent with the results of assays determining contents of organic components, i.e., carbohydrates, lignin and soluble substances. For bark the same trend for changes in the percentage composition (i.e., a change in the percentage content with a change in stress intensity) was detected for three out of six components (i.e., for cellulose, lignin and substances soluble in EtOH), while for wood it was for only one component, holocellulose. This indicates a greater sensitivity of bark to the effect of environmental stress compared to wood.

### 3.2. Sorption Experiments

**Figure 2:**
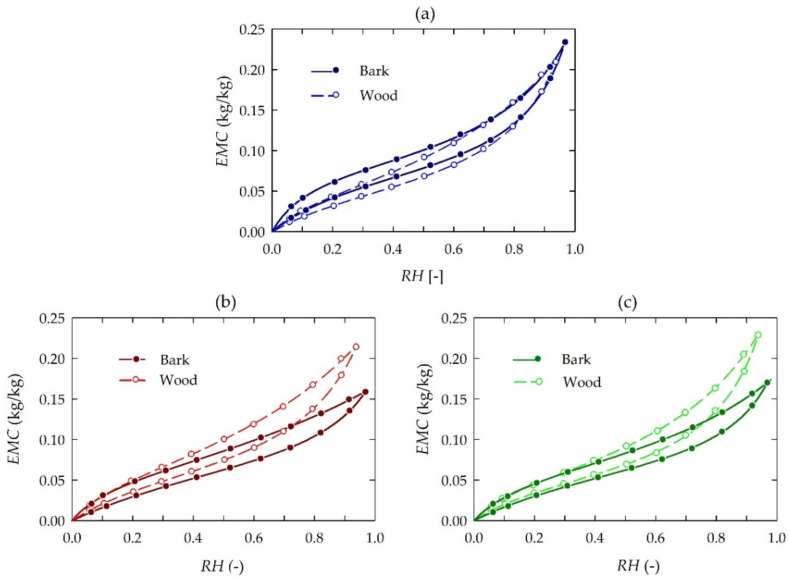
Experimental sorption data (dots) and results of fitting (lines) with the GDW model for *Tilia cordata* Mill. (bark and wood) at 20 °C depending on tree growth conditions: (**a**) low environmental stress, (**b**) medium environmental stress, (**c**) high environmental stress.

The measured *EMC* data are given in Figure 2. Results of the sorption experiments confirm different courses of sorption processes (sorption isotherms) for lime bark in comparison to those of xylem. At the same time, water sorption processes in bark show a markedly greater dependence on the degree of environmental stress when compared to analogous phenomena in xylem. The four-parameter GDW sorption model was separately fitted to bark and wood sorption data. Each plot consists of two sets of adsorption and desorption experimental data, i.e., bark and wood, depending on tree growth conditions. The fitting results for all the wood sample sets are presented in Table 4. The results of sorption experiments generally indicate that the hygroscopicity of bark and wood differ. The slight differentiation of hygroscopicity was in bark and wood of the trees growing under low stress conditions, i.e., in the forest. In this case, the *EMC* absorbed by bark was slightly greater than that of wood for *RH* below 0.60. In the other cases, i.e., regarding medium and high stress conditions, respectively, a greater variety was observed for bark and wood *EMC*. In the trees growing under high stress conditions for *RH* near the saturation point (above 0.90), the bark tissue shows lower *EMC* than wood.

Generally, the estimated values of the *m* coefficient (maximum content of water bound to the primary sites) are higher for an adsorption phase than for a desorption one (see Table 4). Simultaneously, primary water sorption phenomena in lime bark are characterised by considerably higher *m* values than those for wood. The bark of trees growing in high environmental stress areas was characterized by the greatest accessibility of the primary sorption sites (for the desorption phase). The GDW model considers the multilayer sorption depending on the *w* parameter. The physical meaning of the *w* parameter denotes a measure of water molecules bound to the primary sites and converted into the secondary ones. It concerns water absorption and the formation of secondary sorption sites to bind multilayer water. If *w* < 1, then water molecules absorbed on primary sites are not completely converted into secondary sorption sites. If *w* > 1, then each monolayer water molecule is statistically converted into more than one secondary sorption site. For the adsorption phase, the estimated values of the *w* parameter were below one for each lime bark and wood sample. Moreover, the estimated values of the *w* parameter for bark were lower than those for wood. Generally, it can be interpreted as fewer primary sites being converted into secondary ones. This convertibility is lower for bark than for wood. Additionally, the estimated *K* parameter of the GDW model was used to classify sorption isotherms according to the criterion proposed by Furmaniak et al. [38]. In the present study the *K* parameter of the GDW model was higher than unity for each sample option. Therefore, all the isotherms were classified as type II.

**Figure 3:**
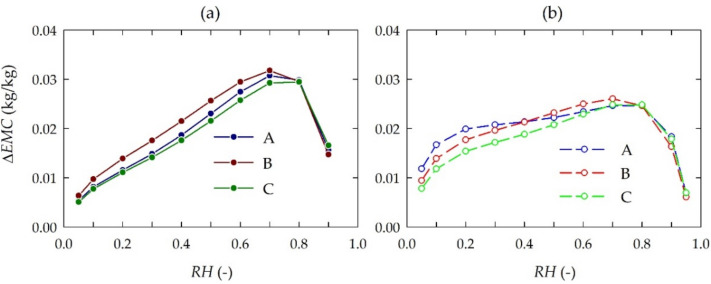
**S**orption hysteresis Δ*EMC* of lime (*Tilia cordata* Mill.): (**a**) bark and (**b**) wood at 20 °C depending on growth conditions: A, B, C—low, medium and high environmental stress, respectively.

The sorption hysteresis are shown in Figure 3 and Table 5. It was found that bark is characterized by a slightly greater maximum difference in *EMC* for desorption and adsorption (Δ*EMC_max_*) compared to wood. Moreover, the greatest differentiation of sorption hysteresis loops was connected with the indices for bark and wood collected from trees growing under low stress conditions. In the other cases, sorption hysteresis loops for bark and wood were similar.

## 4. Discussion

Urbanization, transport and industry are sources of pollution that worsen air quality. Trees are natural air filters that remove pollutants. The *Tilia* tree is one of the tree species that is very common both in parks and along the roads and forests of our climate zone.

Urban greenery has many advantages, one of the most important being the accumulation of CO_2_, the main greenhouse gas. Trees growing in the city contribute to reducing the so-called heat islands, suppress noise, improve climatic conditions and water quality, reduce the leakage of bitumen, contribute to the development of tourism and improve the appearance of the city. The leaves of all the studied *Tilia* species have, to a greater or lesser extent, the ability to accumulate micro-dust pollutants from the air [6,39].

Differences in the chemical composition of *Tilia* wood and bark under different growing conditions indicate the sensitivity of the species to environmental stress. This is consistent with the literature on the subject presenting findings related to other species [40]. Further, the more pronounced degree of differences in the chemical composition of bark than wood suggests that the bark is more sensitive to the environment than the wood. More specifically, changes in the contents of the cellulose, lignin and substances soluble in EtOH of bark of trees growing under three environmental stresses revealed that trees adopt certain physiological mechanisms to cope with the abiotic stresses [41,42,43].

It was observed that the species *Tilia cordata* Mill. increased its resistance to environmental stress through changes in the chemical composition. It was shown that a tree growing under adverse environmental conditions, i.e., in an urban agglomeration, produced more extractives and lignin, providing protective properties. Bark was found to contain more components soluble in EtOH, which serve protective functions and reduce the effect of such negative factors as pollution or droughts. Their protective functions were previously described in the literature [44,45]. In turn, the greatest amounts of lignin, controlling cell–water relations, were recorded in the wood of trees growing under high stress conditions. The importance of lignin as a component protecting against water loss was described, e.g., by Ten and Vermerris [46] and Mahmood et al. [47]. Biosynthesis used for the production of protective substances enhances the attractiveness of *Tilia cordata* Mill. as a species for urban landscaping, since it increases its chances for survival under adverse environmental conditions.

This study showed a dependence between very high contents of potassium and calcium in the bark of the tree growing under low environmental stress and low levels of heavy metals, such as iron, zinc, lead, cadmium and chromium. In wood samples from a tree growing under a high environmental stress, in which very high contents of potassium and calcium were recorded, the highest concentrations of cadmium, nickel and chromium were also detected. A similar trend was observed for lead, zinc, chromium, arsenic and mercury assayed in bark. In the case of the above-mentioned elements, the lowest concentrations were found in each case in bark, while they were the highest in bark from a tree growing under high stress conditions, which may indicate a gradual accumulation of these elements in the bark of lime trees with an increased level of environmental stress. A dependence was observed between the increase in contents of this element in bark depending on the distance from urban areas. In contrast, the results for selected elements assayed in lime wood showed a different trend.

Bark is generally higher in inorganics than wood. The major inorganic elements in bark are Na, K, Ca, Mg and Zn. There is more Na, K, Mg and Zn in wood than in bark, and more Ca in bark than in wood [48]. The inorganic content varies depending on the environmental conditions in which the tree is growing (Saka and Goring 1983). An interaction between Ca and heavy metals in the wood and bark of a two-year-old Norway spruce (*Picea abies* L.) was found by Österås and Greger [49]. Elevated Ca additions decreased the Cd content in bark and the Zn content in old wood, while it tended to decrease the Cu content of bark and the Cd content of old wood. The Ca content decreased in both wood and bark after Cu addition and a high Cd addition. Thus, even small changes in metal availability and proportions in forest soil, such as after spreading of wood ashes in the forest, will be reflected in the metal contents of wood and bark of forest trees.

Another study [50] assessed the health-promoting properties and mineral contents in the bark of bird cherry (*Prunus padus* L.), which was then used as an ingredient in functional teas. Infusions were made from *Matricaria chamomilla* L., *Tilia cordata* Mill. and *Calendula officinalis* L. and then combined with bark in various proportions. The prepared infusions were tested for their antioxidant activity, ability to reduce copper ions and iron ions, as well as the ability to scavenge hydroxyl radicals. Moreover, bird cherry bark contains a high potassium content (ca. 20 mg/kg) [50].

The analyses confirmed considerable differences in the higroscopicity of lime bark and wood—primarily in the case of trees growing under medium and high environmental stress. This may be an indirect effect of growth conditions on the chemical composition of the tested tree tissues. The results of experiments confirmed a significant influence of the chemical composition on the higroscopicity. However, in terms of the effect of air with a higher *RH*, the bark tissue showed lower *EMC* than the wood tissue (except for bark collected from trees growing in the forest). For higher *RH,* the significantly lower *EMC* of bark compared to that of wood was a consequence of significantly lower contents of cellulose and pentosans and a higher content of extractives (see Table 1). The influence of extractives in the sorption was described previously, and it was stated that their high content effected a lower *EMC* [51,52]. The removal of extractives resulted in higher *EMC* as well as in a higher rate of sorption, both in adsorption and in desorption [53]. Hernandez [54] stated that wood containing higher extractives shows low *EMC*, especially when *RH* is above 0.5. Moreover, Spalt [55] pointed out that wood extractives have little effect on monolayer sorption (for low *RH* values), while they have a marked effect on multilayer sorption (for higher *RH*).

## 5. Conclusions

Chemically, *Tilia cordata* Mill bark is very different from wood, presenting much higher levels of substances soluble in NaOH, ethanol and lignin, and a lower polysaccharide content. Growth conditions clearly affect the contents of selected chemical components in bark, e.g., substances soluble in ethanol and in lignin. In relation to wood, bark is characterised by a greater variation in the composition of elements depending on the level of environmental stress. Moreover, the results of sorption experiments confirmed a different course of sorption phenomena (sorption isotherms) in lime bark compared to wood. At the same time, water sorption phenomena in bark show a markedly greater dependence on the level of environmental stress in relation to those in wood. Water sorption phenomena in bark are characterised by a greater sorption hysteresis loop and the maximum difference in equilibrium moisture contents for desorption and adsorption (Δ*EMC_ma_*_x_) compared to those in wood. A lower value in equilibrium moisture content (*EMC*) in bark tissue for high values of relative humidity (*RH*) in the case of trees growing under medium and high environmental stress is a consequence of low contents of cellulose and hemicellulose as well as abnormally high contents of extractives. Results of the sorption analyses based on the parameters of the GDW sorption model indicate that lower *EMC* in bark results from the limitation of multilayer sorption. Biosynthesis directed towards the production of extractives affects hygroscopic properties and increases attractiveness of *Tilia cordata* Mill. as a species used in landscaping in urban agglomerations.

## Figures and Tables

**Table 1:** Chemical composition of lime (*Tilia cordata* Mill.) bark and wood.

Wood Samples		Holocellulose (%)	Cellulose (%)	Pentosans (%)	Lignin (%)	Substances Soluble in NaOH (%)	Substances Soluble in EtOH (%)
Tissue	Environment						
Bark	Low stress	57.1 ^a^ ± 2.1	27.8 ^a^ ± 0.5	13.4 ^a^ ± 0.2	43.0 ^b^ ± 0.1	51.6 ^b^ ± 0.1	14.7 ^b^ ± 0.2
	Medium stress	60.0 ^a^ ± 1.2	30.7 ^b^ ± 0.5	12.3 ^a^ ± 0.6	50.0 ^c^ ± 0.3	44.4 ^a^ ± 0.6	11.8 ^a^ ± 0.4
	High stress	58.3 ^a^ ± 0.8	33.4 ^c^ ± 1.2	12.6 ^a^ ± 0.01	41.0 ^a^ ± 0.1	45.1 ^a^ ± 0.1	16.4 ^c^ ± 0.05
Wood	Low stress	86.1 ^b^ ± 1.1	45.8 ^b^ ± 1.3	22.9 ^a^ ± 1.2	18.7 ^a^ ± 1.1	21.6 ^a^ ± 2.7	6.5 ^a^ ± 1.5
	Medium stress	83.5 ^a^ ± 2.9	42.6 ^a^ ± 1.0	22.7 ^a^ ± 0.8	19.1 ^a,b^ ± 1.3	27.5 ^b^ ± 4.1	10.3 ^b^ ± 2.9
	High stress	84.7 ^a,b^ ± 1.5	43.3 ^a^ ± 1.5	23.3 ^a^ ± 1.4	20.3 ^b^ ± 0.6	25.3 ^b^ ± 1.1	8.1 ^a,b^ ± 0.4

Mean value (*n* = 3) ± standard deviation; identical superscripts (^a^, ^b^, ^c^) denote no significant difference (*p* < 0.05) between mean values according to post hoc Tukey’s HSD test.

**Table 2:** Chemical composition of elements (K, Na, Mg, Ca) in *Tilia cordata* Mill. (bark and wood).

Wood Samples		K (mg/kg)	Na (mg/kg)	Mg (mg/kg)	Ca (mg/kg)
Tissue	Environment				
Bark	Low stress	2170 ^c^ ± 5	2667 ^c^ ± 4	675 ^a^ ± 18	1566 ^a^ ± 307
	Medium stress	2080 ^b^ ± 12	1622 ^a^ ± 12	942 ^c^ ± 8	3442 ^b^ ± 111
	High stress	1910 ^a^ ± 13	1957 ^b^ ± 14	851 ^b^ ± 5	3776 ^b^ ± 9
Wood	Low stress	1222 ^a^ ± 10	1980 ^a^ ± 8	943 ^c^ ± 5	3547 ^a^ ± 57
	Medium stress	1593 ^b^ ± 21	2375 ^b^ ± 7	688 ^b^ ± 6	3935 ^b^ ± 95
	High stress	2360 ^c^ ± 11	1997 ^a^ ± 7	658 ^a^ ± 3	4724 ^c^ ± 24

Mean value (*n* = 3) ± standard deviation; identical superscripts (^a^, ^b^, ^c^) denote no significant difference (*p* < 0.05) between mean values acc. to post hoc Tukey’s HSD test.

**Table 3:** (a) Chemical composition of elements (Fe, Zn, Cu, Pb, Cd) in *Tilia cordata* Mill. bark and wood; (b) Chemical composition of elements (B, Ni, Cr, Al, As, Hg) in *Tilia cordata* Mill. bark and wood.

(a)							
Wood Samples		Fe (mg/kg)	Zn (mg/kg)	Cu (mg/kg)	Pb (mg/kg)	Cd (mg/kg)	
Tissue	Environment						
(b)							
Wood Samples		B (mg/kg)	Ni (mg/kg)	Cr (mg/kg)	Al (mg/kg)	As (µg/kg)	Hg (µg/kg)
Tissue	Environment						
Bark	Low stress	160.5 ^a^ ± 4.4	13.6 ^a^ ± 0.3	5.74 ^b^ ± 0.22	0.050 ^a^ ± 0.01	0.016 ^a^ ± 0.004	
	Medium stress	336.7 ^c^ ± 1.3	15.8 ^b^ ± 0.1	4.59 ^a^ ± 0.01	0.096 ^a^ ± 0.01	0.304 ^b^ ± 0.011	
	High stress	242.2 ^b^ ± 1.1	18.0 ^c^ ± 0.3	5.28 ^b^ ± 0.22	1.626 ^b^ ± 0.21	0.609 ^c^ ± 0.075	
Wood	Low stress	187.3 ^c^ ± 1.7	25.3 ^b^ ± 0.3	9.0 ^b^ ± 0.15	7.96 ^a^ ± 0.27	0.138 ^a^ ± 0.0120	
	Medium stress	115.0 ^a^ ± 0.4	34.5 ^c^ ± 0.1	16.9 ^c^ ± 0.14	26.5 ^c^ ± 0.34	0.48 ^b^ ± 0.010	
	High stress	132.5 ^b^ ± 0.8	24.0 ^a^ ± 0.1	6.8 ^a^ ± 0.17	10.1 ^b^ ± 0.30	0.75 ^c^ ± 0.012	
	Low stress	15.6 ^c^ ± 0.4	0.45 ^a^ ± 0.04	2.42 ^a^ ± 0.14	724 ^b^ ± 3	0.012 ^a^ ± 0.0003	0.025 ^a^±0.0007
Bark	Medium stress	4.3 ^b^ ± 0.5	0.02 ^a^ ± 0.01	3.95 ^b^ ± 0.20	792 ^c^ ± 14	0.015 ^b^ ± 0.0001	0.030 ^b^±0.0001
	High stress	0.5 ^a^ ± 0.1	3.92 ^b^ ± 0.35	7.50 ^c^ ± 0.29	687 ^a^ ± 8	0.018 ^c^ ± 0.0003	0.035^c^±0.0006
	Low stress	19.1 ^a^ ± 0.1	5.70 ^b^ ± 0.20	5.14 ^a^ ± 0.04	787 ^c^ ± 10	0.027 ^b^ ± 0.0003	0.044 ^a^±0.0170
Wood	Medium stress	11.6 ^b^ ± 0.4	0.53 ^a^ ± 0.02	4.88 ^a^ ± 0.19	720 ^b^ ± 6	0.037 ^c^ ± 0.0001	0.073 ^b^±0.0002
	High stress	1.59 ^a^ ± 0.12	5.99 ^c^ ± 0.03	8.79 ^b^ ± 0.10	611 ^a^ ± 3	0.024 ^a^ ± 0.0001	0.041 ^a^±0.0006

Mean value (*n* = 3) ± standard deviation; identical superscripts (^a^, ^b^, ^c^) denote no significant difference (*p* < 0.05) between mean values acc. to *post hoc* Tukey’s HSD test.

**Table 4:** Coefficient coefficients of the sorption GDW model for lime (*Tilia cordata* Mill.) bark and wood.

Wood Samples		Sorption Phase	*m* (kg/kg)	*K*	*k*	*w*	*R* ^2^
Tissue	Environment						
Bark	Low stress	Ads.	0.1017	3.0272	0.8813	0.3594	0.99998
		Des.	0.0931	7.2482	0.7851	0.5883	0.99992
	Medium stress	Ads.	0.1153	1.6339	0.8695	0.2637	0.99992
		Des.	0.0740	5.5129	0.6055	1.2209	0.99995
	High stress	Ads.	0.1292	1.3830	0.8441	0.2566	0.99999
		Des.	0.0655	6.9779	0.4307	2.5057	0.99991
Wood	Low stress	Ads.	0.0811	2.6942	0.8654	0.6059	0.99995
		Des.	0.0564	6.7129	0.6102	2.4967	0.99979
	Medium stress	Ads.	0.0716	3.5722	0.8845	0.6433	0.99985
		Des.	0.0645	6.2429	0.7180	1.5360	0.99990
	High stress	Ads.	0.0849	2.9787	0.8441	0.6397	0.99996
		Des.	0.0681	6.4033	0.5760	2.2986	0.99981

**Table 5:** Sorption hysteresis loops (*H*), maximum differences in *EMC* for desorption and adsorption (Δ*EMC_ma_*_x_) and corresponding relative humidity (*RH*) indices for lime (*Tilia cordata* Mill.) wood.

Wood Samples		*H*	Δ*EMC_max_*	*RH*
Tissue	Environment	(Arb. Units)	(kg/kg)	(-)
Bark	Low stress	0.0194	0.031	0.76
	Medium stress	0.0175	0.030	0.76
	High stress	0.0189	0.032	0.71
Wood	Low stress	0.0179	0.025	0.74
	Medium stress	0.0172	0.025	0.76
	High stress	0.0193	0.026	0.72

## Data Availability

The data presented in this study are available in Supplementary Materials.

## Supplementary Materials

The following supporting information can be downloaded at: https://www.mdpi.com/article/10.3390/ma15114033/s1, Table S1: Two-way ANOVA tables for the chemical composition of lime (*Tilia cordata* Mill.) bark and wood depending on tissue and environment; Table S2: Two-way ANOVA tables for the chemical composition of elements (K, Ca, Na, Mg) in lime (*Tilia cordata* Mill.) depending on tissue and environment; Table S3: Two-way ANOVA tables for the chemical composition of elements (Fe, Zn, Cu, Pb, Cd, B, Ni, Cr, Al, As, Hg) of lime (*Tilia cordata* Mill.) wood depending on tissue and environment.
