# Supplementary material for: Chemical Composition and Related Properties of Lime (Tilia cordata Mill.) Bark and Wood as Affected by Tree Growth Conditions
Source: Materials (Basel). 2022 Jun 6;15(11):4033. doi: 10.3390/ma15114033 (PMC9182011; doi:10.3390/ma15114033)
Supplement: Supplementary file 1 [file materials-15-04033-s001.zip › materials-1722558-supplementary.pdf]

materials MDPI

Table S1. Two-way ANOVA tables for the chemical composition of lime (*Tilia cordata* Mill.) bark and wood depending on tissue and environment.

Chemical composition	Effect	SS	df	MS	F value	p-value
Holocellulose	Tissue (a)	4237.1	1	4237.1	613.67	0.0000
	Environment (b)	1.4273	2	0.71363	0.10336	0.9021
	a x b	81.796	2	40.898	5.9235	0.0058
Cellulose	Tissue (a)	1163	1	1163.2	652.22	0.0000
	Environment (b)	11.156	2	5.58	3.1277	0.0553
	a x b	55.624	2	27.81	15.594	0.0000
Pentosans	Tissue (a)	726.37	1	726.37	564.13	0.0000
	Environment (b)	3.6972	2	1.849	1.4357	0.2512
	a x b	2.0090	2	1.004	0.78013	0.4659
Lignin	Tissue (a)	4504.5	1	4504.5	4095.3	0.0000
	Environment (b)	156.85	2	78.427	71.304	0.0000
	a x b	68.375	2	34.187	31.082	0.0000
Substances soluble in	Tissue (a)	3082.4	1	3082.4	416.14	0.0000
NaOH	Environment (b)	14.376	2	7.1881	0.97044	0.3892
	a x b	168.12	2	84.061	11.349	0.0002
Substances soluble in	Tissue (a)	240.39	1	240.39	58.599	0.0000
EtOH	Environment (b)	68.245	2	34.122	8.3180	0.0010
	a x b	11.611	2	5.8054	1.4152	0.2557

SS – a sum of squares, df – degrees of freedom, MS – mean squares, F – Fisher`s F – test

Table S2. Two-way ANOVA tables for the chemical composition of elements (K, Ca, Na, Mg) in lime (*Tilia cordata* Mill.) depending on tissue and environment.

Chemical composition	Effect	SS	df	MS	F value	p-value
K	Tissue (a)	486267	1	486267	3020.7	0.0000

	Environment (b)	601972	2	300986	1869.7	0.0000
	a x b	1520713	2	760356	4723.3	0.0000
Ca	Tissue (a)	5855263	1	5855263	294.68	0.0000
	Environment (b)	8934733	2	4467367	224.83	0.0000
	a x b	1744419	2	872209	43.896	0.0000
Na	Tissue (a)	5590.2	1	5590.2	67.438	0.0000
	Environment (b)	451614	2	225807	2724.0	0.0000
	a x b	1555546	2	777773	9382.7	0.0000
Mg	Tissue (a)	16011	1	16011	202.38	0.0000
	Environment (b)	13416	2	6707.9	84.792	0.0000
	a x b	243681	2	121841	1540.1	0.0000

SS – a sum of squares, df – degrees of freedom, MS – mean squares, F – Fisher`s F-test.

Contents of potassium, sodium, magnesium and calcium (Table 2) in bark and wood changed markedly and differences between the results were statistically significant.

Table S3. Two-way ANOVA tables for the chemical composition of elements (Fe, Zn, Cu, Pb, Cd, B, Ni, Cr, Al, As, Hg) of lime (*Tilia cordata* Mill.) wood depending on tissue and environment.

Chemical composition	Effect	SS	df	MS	F value	p-value
Fe	Tissue (a)	46393	1	46393	10915	0.0000
	Environment (b)	8730.2	2	4365.1	1027.0	0.0000
	a x b	46489	2	23245	5468.9	0.0000
Zn	Tissue (a)	658.65	1	658.65	13953	0.0000
	Environment (b)	105.32	2	52.662	1115.6	0.0000
	a x b	122.62	2	61.311	1298.9	0.0000
Cu	Tissue (a)	145.29	1	145.29	5085.9	0.0000
	Environment (b)	70.277	2	35.139	1230.0	0.0000
	a x b	100.43	2	50.214	1757.7	0.0000
Pb	Tissue (a)	913.57	1	913.57	15915	0.0000

	Environment (b)	289.38	2	144.69	2520.5	0.0000
	a x b	331.08	2	165.54	2883.8	0.0000
Cd	Tissue (a)	0.09838	1	0.09838	96.296	0.0000
	Environment (b)	1.0965	2	0.54826	536.65	0.0000
	a x b	0.00231	2	0.00115	1.1288	0.3555
B	Tissue (a)	69.862	1	69.862	750.47	0.0000
	Environment (b)	805.62	2	402.81	4327.1	0.0000
	a x b	28.597	2	14.298	153.60	0.0000
Ni	Tissue (a)	30.544	1	30.544	1094.9	0.0000
	Environment (b)	66.639	2	33.319	1194.4	0.0000
	a x b	17.322	2	8.6608	310.45	0.0000
Cr	Tissue (a)	12.198	1	12.198	386.88	0.0000
	Environment (b)	66.769	2	33.384	1058.8	0.0000
	a x b	2.6887	2	1.3443	42.638	0.0000
Al	Tissue (a)	3554.9	1	3555	53.287	0.0000
	Environment (b)	45746	2	22873	342.85	0.0000
	a x b	18625	2	9312	139.59	0.0000
As	Tissue (a)	0.00091	1	0.00091	17590	0.0000
	Environment (b)	0.00011	2	0.00006	1111.8	0.0000
	a x b	0.00018	2	0.00009	1732.1	0.0000
Hg	Tissue (a)	0.00233	1	0.00233	48.328	0.0000
	Environment (b)	0.00091	2	0.00046	9.4590	0.0034
	a x b	0.00110	2	0.00055	11.419	0.0017

SS – a sum of squares, df – degrees of freedom, MS – mean squares, F – Fisher`s F-test.

Contents of selected heavy metals (Tables 3 a, b) determined in bark and wood changed greatly. Differences in the obtained results were statistically significant.
